# Tailored Anti‐miR Decorated Covalent Organic Framework Enables Electrochemical Detection of Salivary miRNAs for Mild Traumatic Brain Injury

**DOI:** 10.1002/smll.202412107

**Published:** 2025-02-17

**Authors:** Pranay Saha, David Skrodzki, Teresa Aditya, Parikshit Moitra, Maha Alafeef, Ketan Dighe, Matthew Molinaro, Steven D. Hicks, Dipanjan Pan

**Affiliations:** ^1^ Department of Nuclear Engineering The Pennsylvania State University University Park PA 16802 USA; ^2^ Department of Materials Science and Engineering The Pennsylvania State University University Park PA 16802 USA; ^3^ Biomedical Engineering Department Jordan University of Science and Technology Irbid 22110 Jordan; ^4^ Department of Biomedical Engineering The Pennsylvania State University University Park PA 16802 USA; ^5^ Department of Engineering Science and Mechanics The Pennsylvania State University University Park PA 16802 USA; ^6^ Department of Pediatrics Penn State Health Children's Hospital Hershey PA 17033 USA; ^7^ Huck Institutes of the Life Sciences 101 Huck Life Sciences Building University Park PA 16802 USA; ^8^ Center for Infectious Disease Dynamics The Pennsylvania State University University Park PA 16802 USA; ^9^ Present address: Department of Chemical Sciences IISER Berhampur Berhampur Odisha 760010 India

**Keywords:** covalent organic framework, electrochemistry, nanoparticle, salivary miRNAs, traumatic brain injury

## Abstract

MicroRNAs (miRNAs) play pivotal role as biomarkers for various diseases, with salivary miRNAs offering a non‐invasive diagnostic tool. For mild traumatic brain injury (mTBI), salivary miRNAs like miR‐let7a, miR‐21, and miR‐30e show promise for early detection of subtle injuries lacking reliable indicators. To advance the detection of mTBI‐related salivary miRNAs, this study integrates anti‐miRNA and miRNA hybridization‐based sensing with the development of a nanoscale covalent‐organic framework (COF) platform. COFs, with their highly customizable structures, large surface area, and biocompatibility, serve as a versatile foundation for biosensing applications. Here, post‐synthetic modification (PSM) of COFs is introduced for essential covalent conjugation of streptavidin for further immobilization of methylene blue‐labeled and biotinylated Anti‐miRNAs. Furthermore, the layer‐by‐layer assembly of conductive polymers enhanced the biosensor's electrical performance, enabling ultrasensitive and multiplexed detection of salivary miRNAs. Validated with samples from mixed martial arts participants and confirmed by polymerase chain reaction (PCR), this COF‐based platform demonstrates robust accuracy and reliability. By combining COF functionalization with advanced electrode design, it offers a powerful, non‐invasive solution for early mTBI detection and broader biomedical applications.

## Graphical abstract

Salivary microRNAs (miRNAs) serve as promising non‐invasive biomarkers for mild traumatic brain injury (mTBI). This study introduces a nanoscale covalent‐organic framework (COF)‐based biosensor for ultrasensitive and multiplexed detection of miRNAs. Functionalized COFs enable efficient anti‐miRNA immobilization, enhancing electrochemical detection. The platform, validated with clinical samples, offers a reliable and rapid tool for early mTBI diagnosis.

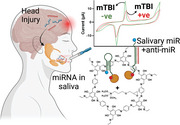

## 1 Introduction

Mild traumatic brain injury (mTBI) is a common neurological injury causing somatic, cognitive, and sleep‐related symptoms, with diagnosis hindered by subtle signs and a lack of reliable biomarkers. The centers for Disease Control and Prevention (CDC) estimates 1.7–3.8 million TBIs annually in the U.S., with over 21% in children and adolescents linked to sports. Classified by the Glasgow Coma Score (GCS), moderate‐to‐severe TBIs need urgent care, while mTBI often goes undiagnosed despite its ties to headaches, fatigue, and missed activities, adding to the healthcare burden.^[^ ^1^ ^]^ The physical, cognitive, and psychological impacts of mTBI necessitate reliable and objective biomarkers for effective diagnosis and treatment. The current gold standard for mTBI diagnosis relies heavily on clinical assessment.^[^ ^2^, ^3^ ^]^ Neuroimaging is rarely used for mTBI due to cost, lack of portability, and difficulty detecting subtle brain changes. Early diagnosis is essential to improve outcomes, but mTBI is often underdiagnosed due to delayed symptoms.^[^ ^4^ ^]^ The 2018 CDC guidelines recommend symptom scales and computerized testing but advise against biomarker use outside research. This highlights the need for non‐invasive, sensitive, and specific diagnostic tools to enable timely intervention, especially in the acute phase.^[^ ^5^ ^]^

Various technologies for TBI diagnosis have been explored, including computerized cognitive assessments, neuroimaging, electrophysiology, and serum protein biomarkers.^[^ ^6^, ^7^, ^8^, ^9^, ^10^, ^11^ ^]^ Computerized assessments are time‐consuming, unreliable for retesting, and unsuitable for young populations.^[^ ^12^, ^13^, ^14^ ^]^ Neuroimaging and electrophysiological methods show weak reliability and require costly, non‐portable equipment. Blood tests by companies like Banyan Biomarkers and Abbott target TBI biomarkers like S100 calcium‐binding protein B (S100B), glial fibrillary acidic protein (GFAP), ubiquitin carboxy‐terminal hydrolase L1 (UCH‐L1) but remain unvalidated for those under 18, lack long‐term predictive value, and are impractical in field settings without venipuncture training.^[^ ^15^, ^16^ ^]^ While promising in adults, serum protein biomarkers are unvalidated in those under 18, may not predict long‐term outcomes, and are impractical for use on battlefields or sports without venipuncture training.^[^ ^11^, ^17^, ^18^ ^]^ A simple, accessible biomarker panel is needed for accurate mTBI diagnosis, with fluid‐based proteins or miRNAs offering a promising option for preliminary detection.

MicroRNAs (miRNAs) have emerged as promising biomarkers for mTBI due to their dysregulation in response to neuronal injury and their presence in various biofluids, including saliva.^[^ ^19^, ^20^, ^21^, ^22^ ^]^ These small non‐coding RNA molecules play critical roles in post‐transcriptional gene regulation and are known to be involved in multiple physiological and pathological processes ().^[^ ^23^, ^24^, ^25^, ^26^ ^]^ With their potential for rapid and non‐invasive detection, salivary miRNAs represent a promising tool for improving mTBI diagnosis.^[^ ^27^, ^28^ ^]^ Advances in detection techniques could improve mTBI diagnosis and management, enhancing patient care. Specific miRNA signatures, including miR‐let7a, miR‐21, and miR‐30e, have been identified as differentially expressed in saliva from mTBI patients.^[^ ^29^, ^30^, ^31^, ^32^ ^]^ Saliva, a readily available non‐invasive biofluid, provides a convenient medium for miRNA‐based diagnostics, avoiding invasive procedures. miR‐30e, miR‐21, and let‐7a were selected as mTBI biomarkers after rigorous evaluation of their differential expression in patients compared to healthy controls.^[^ ^33^ ^]^ These miRNAs were selected for their significant dysregulation in mTBI patients, indicating involvement in its pathophysiology. Literature links miR‐30e to neuronal apoptosis, miR‐21 to inflammation, and let‐7a to neurogenesis and survival, highlighting their diagnostic potential for a non‐invasive salivary mTBI test.^[^ ^34^, ^35^, ^36^ ^]^ Standard miRNA profiling techniques like northern blotting, reverse transcription polymerase chain reaction (RT‐PCR), and next‐generation sequencing (NGS) have limitations for rapid, cost‐effective, and accurate mTBI diagnosis due to complexity, radioactive labeling, and high costs.^[^ ^37^, ^38^, ^39^, ^40^, ^41^, ^42^ ^]^ miRNA detection requires high sensitivity and specificity, but small size and RNase susceptibility pose challenges.

**Scheme 1:**
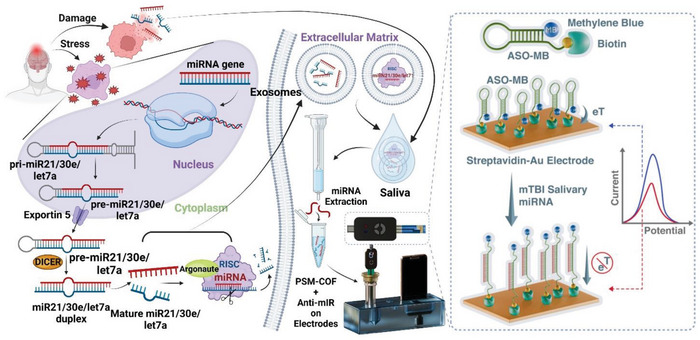
Schematic representation on post‐concussion passivation of miRNAs into saliva through different pathways and the detection procedure of these salivary miRNAs with electrochemical sensing platform for mild traumatic brain injury (mTBI)

The regulation of salivary miRNAs following brain injury is complex and influenced by factors like cellular responses to injury, inflammation, and apoptosis. Systemic effects and blood‐brain barrier disruption further contribute to changes in miRNA expression.^[^ ^43^ ^]^ Post‐concussive miRNA production and their extravasation in saliva is schematically explained in **Scheme** **1**. Studies have identified specific miRNAs, like miR‐21, miR let7a, and miR‐30e, that are upregulated in saliva after brain injury, while others, like miR‐148b and miR‐27a, are downregulated.^[^ ^36^, ^44^, ^45^, ^46^ ^]^ These miRNAs can be transported through the blood‐brain barrier and into the circulatory system, eventually reaching the salivary glands. The extent of up‐and down‐regulation can vary depending on individual factors and the nature of the injury.^[^ ^47^ ^]^ Complementary anti‐miRNA oligonucleotides were designed and immobilized on covalent organic frameworks (COFs) to utilize salivary miRNA signatures for diagnostics. COF‐based conductive electrodes provide stable anti‐miRNA attachment for sensitive electrochemical detection.

Nanoscale covalent organic frameworks (COFs) offer high surface area, tunable porosity, and chemical stability, making them ideal for drug delivery, sensing, and catalysis. Post‐synthetic modification (PSM) introduces functional groups like targeting moieties or therapeutic agents, creating multifunctional theranostic platforms. PSM enhances COF versatility by enabling tailored properties and bioactive molecule incorporation.^[^ ^48^, ^49^, ^50^, ^51^, ^52^, ^53^, ^54^ ^‐^ ^59^ ^]^ Future directions for COFs include investigating multimodal theranostics, increasing functional groups, and enhancing biocompatibility.

**Figure 1:**
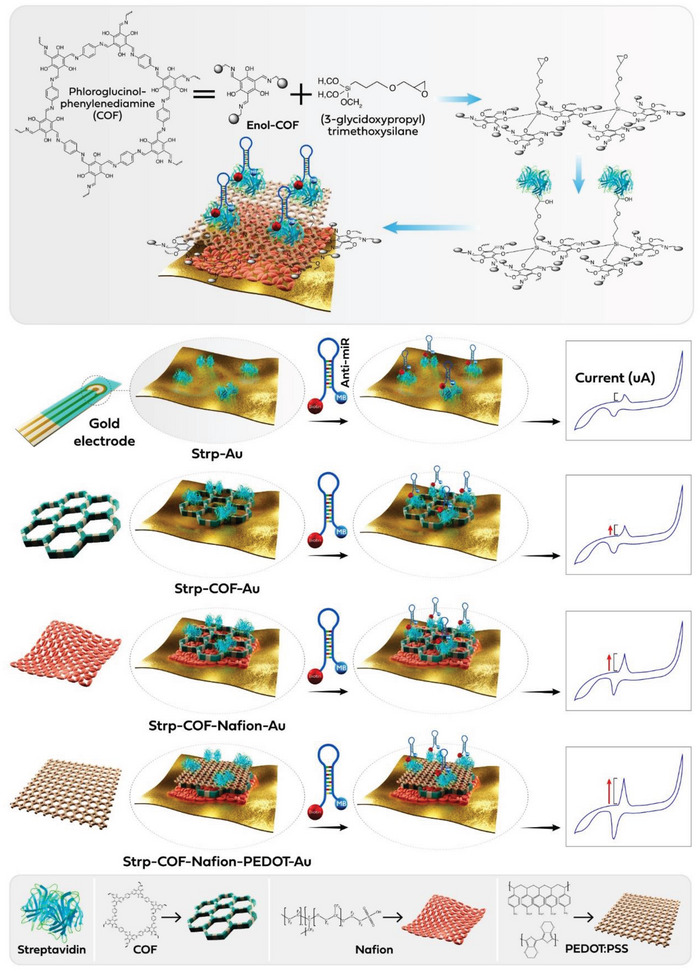
Post‐synthetic modification of Covalent Organic Framework (COF) for enhanced functionalization over Au‐electrodes with Streptavidin and Biotinylated anti‐miR oligonucleotides (top). Layered assembly of streptavidin with the anti‐miRNA oligonucleotide on gold electrodes with post synthetically modified‐COF, Nafion, and PEDOT:PSS enhances electrochemical current output in cyclic voltammetry (bottom).

This work introduces a COF‐based electrochemical biosensor for ultrasensitive detection of mTBI‐specific salivary miRNAs. The biosensor uses a COF based on p‐phenylenediamine and 1,3,5‐triformylphloroglucinol, modified with (3‐glycidoxypropyl)trimethoxysilane (GPTMS) to add epoxy groups for streptavidin immobilization. **Figure** **1** illustrates the stepwise conjugation of GPTMS, streptavidin, and anti‐miR oligos on COF‐modified Au‐electrodes. This functionalization enables stable attachment of methylene blue‐labeled and biotinylated anti‐miRs for targeting specific miRNAs, leveraging oligonucleotide‐based biosensors' high specificity and accuracy.^[^ ^60^, ^61^, ^62^, ^63^, ^64^, ^65^, ^66^, ^67^, ^68^, ^69^ ^]^ To enhance conductivity and sensitivity, conductive layers of Nafion and poly(3,4‐ethylenedioxythiophene) polystyrene sulfonate (PEDOT:PSS) were used (Figure 1, bottom). Nafion provides stability and conductivity, while PEDOT improves electrical properties and dispersion. ASO‐functionalized COFs are deposited on gold electrodes with a Nafion/PEDOT:PSS bilayer, boosting electrochemical signal transduction during miRNA hybridization. This combination offers significant advantages for electrode modification.^[^ ^70^, ^71^ ^]^ This sensor detects picomolar levels of mTBI‐related miRNAs (miR‐let7a, miR‐21, miR‐30e) in saliva.^[^ ^36^, ^72^, ^73^, ^74^ ^]^ PSM in COF enables covalent streptavidin conjugation for biotinylated anti‐miRNA attachment (methylene blue as a reporter) through host‐guest chemistry. The phenylenediamine and 1,3,5‐triformylphloroglucinol‐based COF offers high surface area, stability, and biocompatibility, supported by PEDOT:PSS and Nafion for efficient signal transduction. This saliva‐based biosensor provides a sensitive, non‐invasive platform for early mTBI detection, addressing a key need in neurological care.

## 2 Results and Discussion

### 2.1 Selection of Target miRNAs for mTBI (Mild Traumatic Brain Injury) and Design of Single‐Stranded Anti‐miRNA Oligos

The selection of target miRNAs for mTBI in human saliva involves identifying miRNAs that are pathophysiologically relevant to brain injury, detectable in saliva and exhibit high diagnostic accuracy and stability. Among the 31 miRNAs analyzed, miR‐30e, miR‐21, and let‐7a were selected based on specific criteria. miR‐30e (sequence: UGU AAA CAU CCU UGA CUG GAA G) is involved in neuronal apoptosis and stress responses, miR‐21 (sequence: UAG CUU AUC AGA CUG AUG UUG A) plays a role in inflammation and apoptosis regulation and let‐7a (sequence: GGU AGU AGG UUG UAU AGU U) regulates cell differentiation and proliferation.^[^ ^36^ ^]^ Designing complementary single‐stranded oligonucleotides (ASOs or antisense oligonucleotides) or anti‐miRNAs (anti‐miRs) against these miRNAs involves ensuring sequence specificity and optimal thermodynamic stability. The anti‐sense oligos were designed to incorporate chemical modifications for host‐guest conjugation and generate electrochemical signals upon hybridization with complimentary miRNAs (**Scheme 1**). The oligos were fabricated with methylene blue (MB) and biotin modifications at their opposite ends to enhance electrochemical and photophysical specificity, stability, and host‐guest‐based binding affinity. This approach leverages miRNAs' stability and diagnostic potential in saliva to develop non‐invasive tools for mTBI diagnosis. Biotin can bind to Streptavidin, which can be immobilized on the electrodes, and methylene blue acts as a redox reporter upon conformational or intramolecular distance‐dependent changes. The details of miRNA sequences and anti‐miR oligonucleotide sequences are provided in **Tables** **1** and **2**.

**Table 1:** Complementary anti‐miRNA nucleotide sequences as detection probes.

Anti‐miR	Sequence
let‐7a (miR‐let‐7a‐5p)	MB‐AACTATACAACCTACTACCTCA‐Biotin
miR‐30e (miR‐30e‐5p)	MB‐CTTCCAGTAAGGATGTTTACA‐Biotin
miR‐21 (miR‐21a‐5p)	MB‐TCAACATCAGTCTGATAAGCTA‐Biotin

**Table 2:** Nucleotide sequence database for the miRNAs being diagnosed.

miRNA	Sequence
let‐7a (miR‐let‐7a‐5p)	UGA GGU AGU AGG UUG UAU AGU U
miR‐30e (miR‐30e‐5p)	UGU AAA CAU CCU UGA CUG GAA G
miR‐21 (miR‐21a‐5p)	UAG CUU AUC AGA CUG AUG UUG A

### 2.2 Validation and Specificity of Anti‐miRNA Oligos with Spectroscopy (UV–vis and Fluorescence)

**Figure 2:**
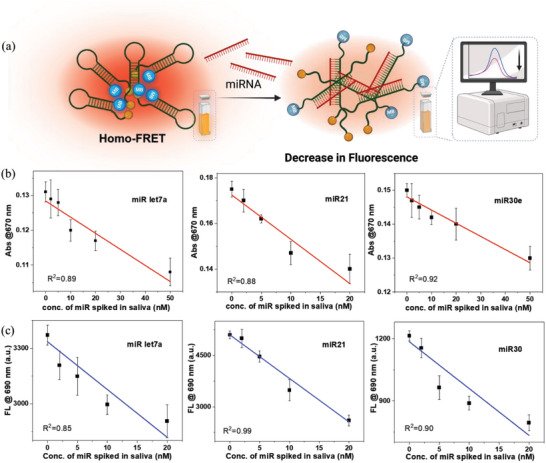
a) Schematic representation of change in the fluorescence of methylene blue‐miR‐ASO upon hybridization with corresponding miRNAs b) Change in absorbance of MB‐miR‐ASO in saliva upon addition of an increasing amount of respective miRNA exhibits linear decrease c) Change in fluorescence of MB‐miR‐ASO in saliva with increasing concentrations of respective miRNA shows similar decrease in fluorescence intensity.

The specificity of MB‐tagged ASOs against their corresponding miRNAs was validated by spectroscopic measurements performed in aqueous medium and artificial saliva. The quenching of fluorescence has been explained schematically in **Figure** **2**a. Artificial saliva was used to evaluate its impact on photophysical measurements, revealing minimal effects in both solution‐phase cuvettes and electrochemical detection on electrodes. A gradual decrease in methylene blue (MB) absorbance (670 nm) and fluorescence (690 nm) was observed during hybridization with increasing concentrations of miRNAs (miR let‐7a, miR‐21, miR‐30e) in artificial saliva. This decrease is attributed to the conformational changes in MB‐tagged antisense oligonucleotides (ASOs) upon binding with complementary miRNAs, altering the MB molecules' electronic environment.^[^ ^75^, ^76^, ^77^ ^]^ The conformational change brings MB closer to the miRNA, facilitating quenching interactions that reduce absorbance at 670 nm (Figure 2b). Hybridization also alters the local environment, including polarity and viscosity, around MB molecules, affecting electronic transitions and further decreasing absorbance. Hybridization induces both static and dynamic quenching of MB fluorescence. Static quenching arises from non‐fluorescent complexes formed between MB and the miRNA‐ASO hybrid, while dynamic quenching involves increased non‐radiative decay due to MB's closer proximity within the hybrid structure. Additionally, Förster Resonance Energy Transfer (FRET) may contribute, as MB's excited‐state energy is transferred to nearby MB molecules, with fluorescence intensity affected by differential distances in the hybrid complex (Figure 2a). A concentration‐dependent decrease in absorbance and fluorescence was observed in artificial saliva and aqueous media (Figure 2b,c; Figure S1, Supporting Information). This phenomenon highlights the potential of MB‐tagged anti‐miR ASOs for electrochemical detection, as described later. Raman spectroscopy confirmed successful ASO‐miR hybridization in solution and on Au electrodes. Anti‐miR and miR interactions on the Au electrode showed changes in surface‐enhanced plasmon response, as the hairpin miR‐ASO structure stretched upon hybridization with complementary miR (Figure S2, Supporting Information), validating the hybridization process.

### 2.3 Synthesis of COF and Characterization

**Figure 3:**
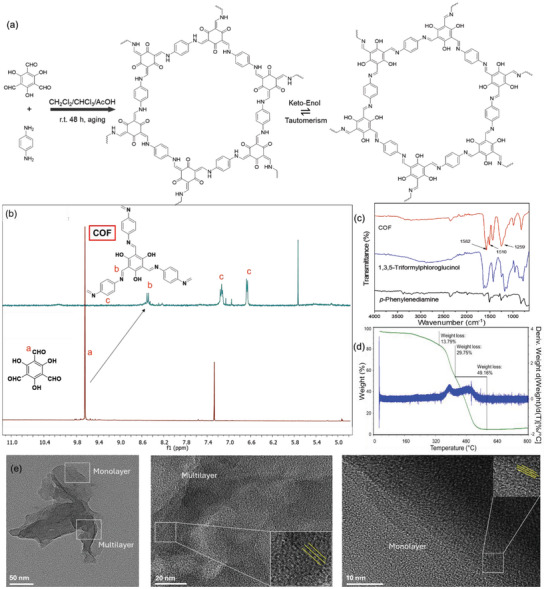
a) Synthesis scheme for phloroglucinol and keto form of the covalent organic framework in organic nonpolar solvent b) change in ^1^H NMR peak on aldehyde to hydroxyl transformation upon keto to enol tautomerization of COF from due to change in solvent from chloroform to DMSO c) FTIR peaks of specific bonds exhibit formation of covalent organic framework from the monomers d) Thermogravimetric analysis shows weight loss of the COF from 22 °C to 800 °C with high stability till 350 °C e) Transmission electron microscopic images showing monolayer and multilayer accumulation of the silane‐modified COF nanosheets with faintly visible lattice fringes.

1,3,5‐Triformylphloroglucinol was synthesized as per previously reported literature and has been described in detail in materials and methods (Figure S3a, Supporting Information).^[^ ^78^ ^]^ 1,3,5‐Triformylphloroglucinol was dissolved in a mixture of dichloromethane (CH₂Cl₂), chloroform (CHCl₃), and acetic acid. The resulting homogeneous solution was maintained at room temperature for 48 h, yielding nanosheet‐like covalent organic frameworks (COFs) at the nanoscale level. The choice of the solvent system (CH₂Cl₂ and CHCl_3_) was critical in achieving the desired morphology. These solvents ensured the complete dissolution of triformylphloroglucinol, facilitating a slow polymerization, nucleation, and growth process that favored the formation of multilayer COF sheets and in contrast, other solvents partially dissolved triformylphloroglucinol, leading to irregular or aggregated particles after the Schiff base reaction. Free hydroxyl groups were required for further functionalization of the COF nanosheets for covalently attaching epoxysilane to attach streptavidin on the COF surface. To promote keto‐enol tautomerism in these COF nanosheets, a few changes were performed regarding solvent, pH, and temperature (**Figure** **3**a). At slightly lower temperatures, COF nanosheets were dispersed in water containing 5% dimethylformamide, and a minimal amount of NaOH was added to make the solvent slightly alkaline. As the keto form is more stable in higher temperatures, lower pH, and nonpolar solvents, reversing these conditions induces an enol form of COF with flanking hydroxyl groups for further conjugation (Figure 3a).

The ^1^H NMR spectra in Figure 3b depict the enol form of the covalent organic framework. In phloroglucinol, the singlet aldehyde proton peak comes within 9–10 ppm, which is visible in NMR. After forming the enol form of COF, proton peaks near 6.6 ppm, and 8.7 ppm denotes the hydroxyl and other benzene ring protons where the aldehyde proton is entirely absent. Aldehyde protons appeared as singlets because there was no neighboring proton with which to couple, but the broadening of peaks in enol‐COF was observed due to hydrogen bonding and proton exchange processes. In Figure S3 (Supporting Information), the XRD showed the COF's crystalline nature, similar to the previously reported article.^[^ ^79^ ^]^ Sharp peaks in the lower 2θ range (2‐10 degrees) correspond to the (100), (110), (200) planes of the nano‐framework structure. The broader peaks correspond to smaller repeat distances within the COF structure and represent the stacking of layers or in the nanosheet structure. The XPS analysis of COF provided information on the C1s, N1s, and O1s components as given in full spectra in Figure S4a (Supporting Information). High‐resolution XPS analysis provided details on the bonding information of the nano‐framework, similar to previous literature.^[^ ^79^ ^]^ For the C1s spectra, the peak at 284.6 eV represents C─H bonds (backbone), 286.02 eV corresponds to C─N bonds, and 288.3 eV to C═O bonds. In the N1s spectra, peaks at 399.4 eV and 400.1 eV indicate C═N and C─N bonds (imine tautomerism), with a π‐π satellite peak at 403.6 eV. The O1s spectra show peaks at 530.7 eV for C═O, 532.6 eV for C─O, and 535.7 eV for O─H, consistent with the COF framework structure. In Figure 3c, the typical C─N and C═C vibrations appeared at 1259 and 1582 cm^−1^, respectively, in the Fourier transform infrared (FT‐IR) spectra, indicating the formation of a b‐ketoenamine‐linked structure.^[^ ^79^ ^]^ The FTIR peak at 1510 cm⁻¹ in p‐phenylenediamine corresponds to C═C stretching vibrations in the aromatic ring. This characteristic vibration, observed in the 1450–1600 cm⁻¹ range, is influenced by para‐positioned amine groups (─NH₂), confirming the benzene ring's presence in the compound. In Figure 3d, thermogravimetric analysis (TGA) shows the COF's high thermal stability, with gradual weight loss from 320 to 400 °C and rapid decomposition in two steps between 400 and 480 °C. While high thermal stability is unnecessary for biochemical sensing, the decomposition profile aligns with regular crystalline COFs, confirming successful synthesis.^[^ ^48^, ^80^, ^81^ ^]^ Changes in thermal stability, as seen in the TGA profile, can also indicate successful modification of the COF nanosheets^[^ ^82^, ^83^, ^84^ ^]^ The TEM images exhibited the mono and multilayer nanosheet structures with visible lattice fringes for both unmodified and silane‐modified COF nanosheets (Figure 3e; Figure S4b, Supporting Information). Enol‐COF was imaged using TEM after dispersing it in a slightly basic aqueous solution and drop‐casting onto grids. The images revealed single and overlapping nanosheets (50–200 nm) with a nanoflake‐like structure, suitable for coating Au electrodes and providing a larger surface area for modification.

### 2.4 Characterization and Standardization of Post‐Synthetic Modification of COF Over Electrode Surface for Conjugation of Anti‐miRNA Oligonucleotides

**Figure 4:**
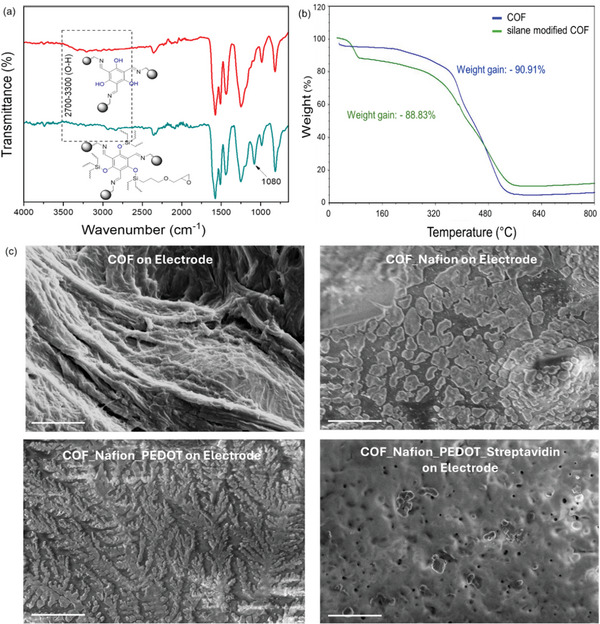
a) FTIR spectra exhibits disappearance of ─OH vibrations around 2700–3300 cm^−1^ after functionalization with GPTMS and new peaks at 1080 cm^−1^ for new C─O bonds generated from GPTMS b) TGA shows differential pattern in weight loss from unmodified COF proving successful modification of the COF with GPTMS c) SEM images of Au electrodes after each level of functionalization shows traces of frameworks, polymers and protein (streptavidin) [scale: 5 µM].

**Figure 5:**
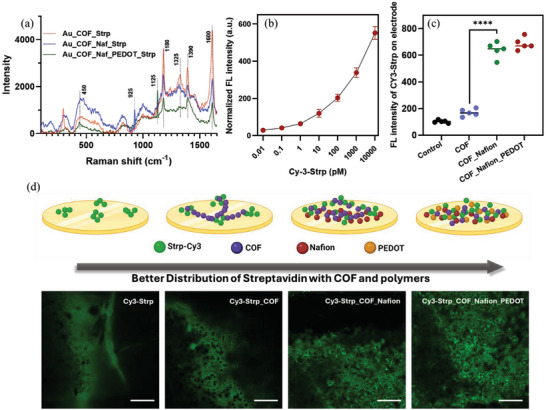
a) Change and shift in Raman peak positions and intensities b) Standard curve for Cy‐streptavidin conjugation on functionalized electrode to determine the concentration of streptavidin attachment over the COF‐Nafion‐PEDOT‐based electrode as depicted from solid state fluorescence spectroscopy c) Differential conjugation profile of Cy3‐streptavidin over the different layers of functionalized electrodes d) Confocal microscopy images exhibit differential distribution pattern of Cy‐3 streptavidin over the modified electrode surface (scale: 50 µM).

The COF was initially characterized using XPS, XRD, NMR, and FTIR spectroscopy, confirming successful fabrication as per previously reported literature.^[^ ^79^ ^]^ FTIR, TGA, SEM, and Raman spectroscopy were used to characterize the electrode surface layers after each fabrication step, confirming successful deposition and functionalization. In the FTIR spectra (**Figure** **4**a), the emergence of a new peak at 1080 cm⁻¹ indicates the formation of a C─O bond from silane modification. The disappearance of ‐OH vibrations ≈ 2700–3300 cm⁻¹ after GPTMS functionalization confirms the successful modification of flanking ‐OH groups as they are replaced by GPTMS.^[^ ^79^ ^]^ TGA analysis (Figure 4b) comparing unmodified (blue curve) and modified (green curve) COFs reveals distinct thermal behavior. Both COFs decompose ≈ 100 °C, with the modified COF showing earlier, gradual weight loss (Figure S5, Supporting Information), indicating potential changes in stability or volatile components from modification. Between 160 °C and 400 °C, the modified COF exhibits gradual weight loss, suggesting enhanced thermal stability. At ∼500 °C, both COFs decompose significantly, but the modified COF retains more weight, indicating improved thermal stability. SEM (Figure 4c) shows distinct surface topologies: plate‐like COF surfaces, fibrous polymer traces, and bumpy membrane‐like protein structures. Raman spectra (**Figure** **5**a) show shifts in key peaks: 450 cm⁻¹ (COF‐gold interaction), 925 cm⁻¹ (SO₃⁻ stretching of Nafion), 1125 cm⁻¹ (C─O‐C in PEDOT:PSS), 1325 cm⁻¹ (C─N or C═C stretching), 1390 cm⁻¹ (C─H or C─N), and 1600 cm⁻¹ (C═C stretching)^[^ ^85^, ^86^ ^]^ Phenylenediamine and 1,3,5‐triformylphloroglucinol‐based COF were modified with (3‐glycidoxypropyl)trimethoxysilane for streptavidin immobilization. The fabrication on carbon and gold electrodes favored Au‐electrodes due to better electrical outputs for a portable potentiostat.

To confirm successful streptavidin conjugation to the COF surface, fluorescent Cy3‐streptavidin (Cy3‐Strp) was applied to freshly prepared epoxy‐functionalized COF‐Au electrodes. Using a solid‐state sample holder and adjusting it to a 60° angle toward the detector in a Horiba Fluromax fluorescence spectrophotometer, the fluorescence signal from the electrodes was measured to correlate with fluorophore density. A standard curve was generated by varying Cy3‐Strp concentrations, determining 20 µg mL^−1^ as optimal (Figure 5b). Unmodified electrodes showed fluorescence below detection limits, while epoxy‐labeled COF surfaces demonstrated clear Cy3‐Strp deposition, indicating effective conjugation (Figure 5c). This density was significantly higher than bare Au electrodes. Confocal microscopy (Figure 5d) revealed that additional Nafion and PEDOT:PSS layers improved Cy3‐Strp distribution over the COF‐Au surface, as shown schematically in Figure 5d.

### 2.5 Electrochemical Detection of miRNAs via Cyclic Voltammetry and Differential Pulse Voltammetry

**Figure 6:**
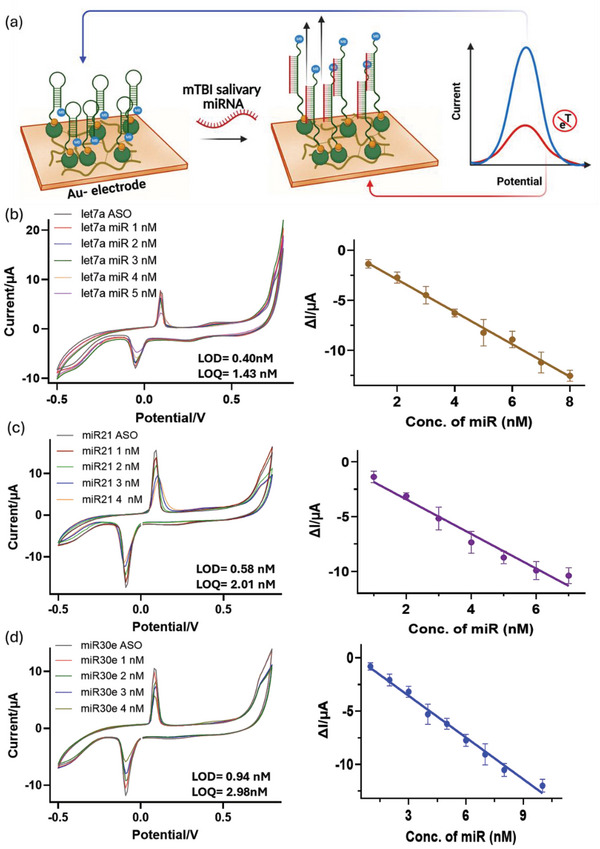
a) Scheme exhibits the mechanism of change in electrical output: Change in cyclic voltammetry current output over COF‐Nafion‐PEDOT‐ASO‐Streptavidin platform over Au electrode upon addition of b) miR let7a c) miR21 d) miR30 exhibits linear change in current with increasing concentrations of miRs.

We demonstrated the use of cyclic voltammetry (CV) and differential pulse voltammetry (DPV) for the detection process (**Figure** **6**), as both techniques are well‐studied for electrochemical detection.^[^ ^87^, ^88^, ^89^, ^90^, ^91^, ^92^ ^]^ CV and DPV were conducted for specific miRNAs (miR‐30e, miR‐21, let‐7a) using MB‐labeled antisense oligonucleotide (ASO) probes conjugated to phloroglucinol‐based COFs. The COFs were modified with GPTMS to introduce epoxy groups for streptavidin immobilization, enabling binding to biotinylated ASOs. Electrochemical parameters are detailed in the experimental section, and the fabrication process was described previously. Hybridization of single‐stranded oligonucleotides does not change the overall charge but alters charge distribution. Negatively charged phosphate groups, exposed in single strands, become partially buried in the double helix, increasing electrostatic repulsion. This change affects guanine base accessibility and electron transfer properties, detectable by cyclic voltammetry (CV) as variations in oxidation peak current and potential.^[^ ^93^ ^]^

The hybridization of miR and anti‐miR oligos was indirectly detected by monitoring changes in CV signals of guanine oxidation and electron transfer.^[^ ^94^, ^95^, ^96^, ^97^ ^]^ MB attached to *ant‐miR* ASOs moved away from the electrode upon miRNA binding, reducing electron transfer efficiency and current (Figure 6a). A gradual decrease in current (ΔI) was observed with increasing miRNA concentrations for miR‐30e, miR‐21, and let‐7a (Figure 6b–d). For miR‐21, the current decreased linearly, with an LOD of 0.4‐1 nM and LOQ of 1.4‐3 nM. Similar trends were noted for all three miRNAs, with slight peak shifts. In DPV, a linear current increase at low concentrations (5‐80 nM) was observed, attributed to potential pulses enhancing current. A new peak suggested a redox‐active ASO‐MB‐miRNA complex (Figure S6a, Supporting Information). However, after multilayer electrode fabrication, DPV signals were less responsive at low concentrations than CV. Also, for miR21, the differential pulse voltammetry results were insignificant (Figure S6b, Supporting Information). DPV offered good sensitivity but was less effective with increased surface activity, making CV the preferred technique. Enhanced electrode functionalization improved electron transfer kinetics and active surface area, enabling stronger miRNA‐ASO interactions and more pronounced redox signals in CV. Consequently, further assessments were conducted using CV only. The complex COF‐polymer‐anti‐miR‐based platform coupled with CV measurements demonstrated excellent selectivity and recovery in detecting these three miRNAs. Selectivity was validated through experiments using non‐specific targets (random oligonucleotides, miR‐320b and miR‐16‐5p), which showed no significant current output, confirming the strong hybridization affinity between the anti‐miRNA probe and its complementary target.^[^ ^94^, ^98^ ^]^ Recovery was assessed by spiking known concentrations of miRNAs into artificial saliva, yielding recoveries ranging from ∼96% to 103%, highlighting the sensor's accuracy and minimal interference from the matrix and similar analytes.^[^ ^94^ ^]^ These findings affirm the sensor's reliability for miRNA quantification in complex biological samples.

### 2.6 Efficacy of Nafion/PEDOT with COF‐Streptavidin System

**Figure 7:**
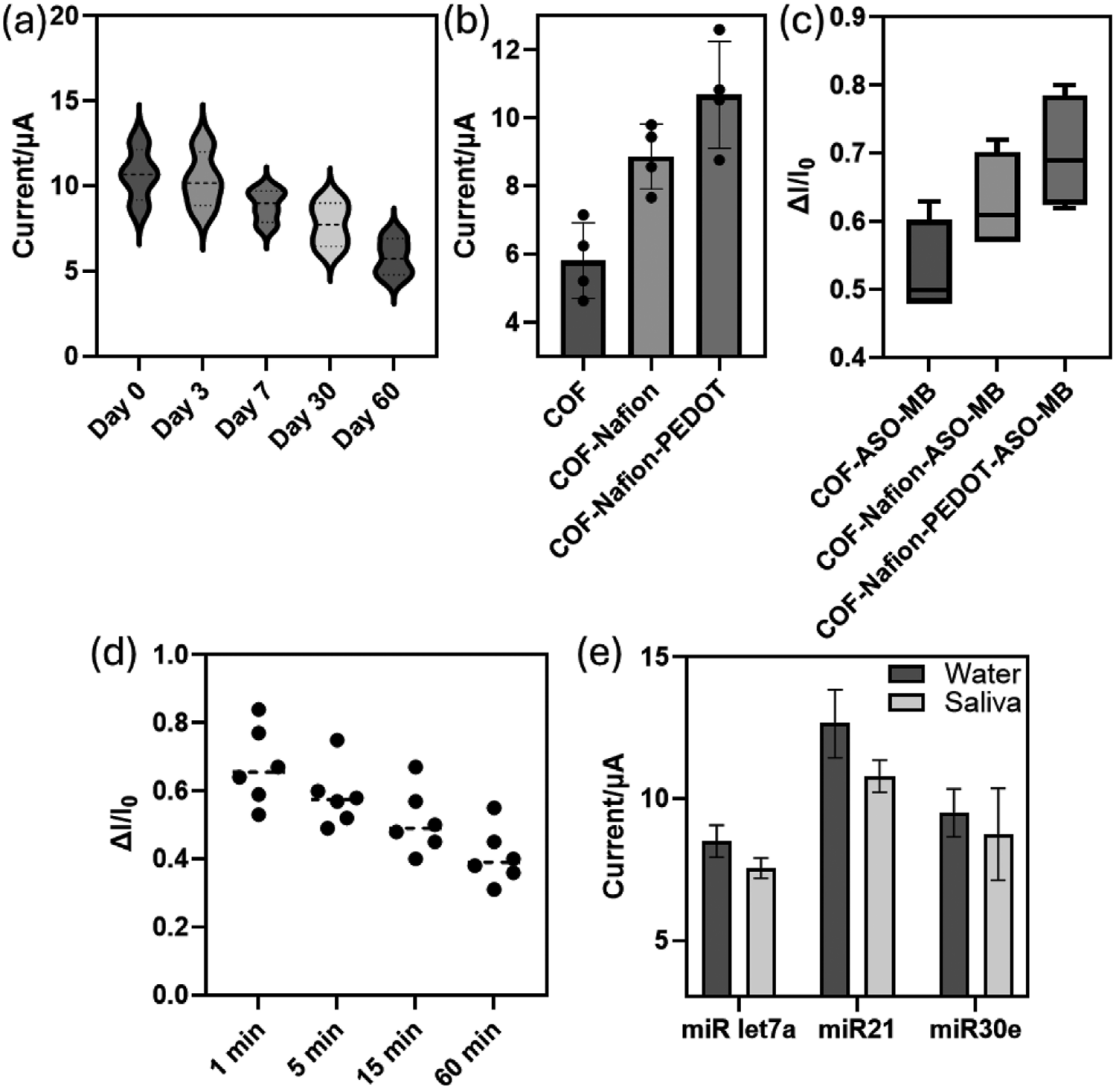
a) Long‐term stability of the COF‐Nafion‐ASO‐MB electrodes up to 60 days b) Increased current output of the electrodes upon functionalization with polymer layers c) Increased ionic conductivity with addition of polymer layers upon hybridization of anti‐miR and miR d) Consistent current output on COF‐Nafion‐PEDOT‐ASO‐MB up to 60 min after hybridization between the anti‐miR and miRNAs e) Saliva shows minimal matrix effect in Cyclic Voltammetry which does not affect the measurements significantly.

Nafion and PEDOT:PSS were integrated with the COF layer in electrode strips to enhance cyclic voltammetry (CV) signals, crucial for detecting low salivary miRNA levels in mild traumatic brain injury (mTBI) patients. Nafion, a sulfonated fluoropolymer, was mixed with COF to improve ionic conductivity, mechanical strength, and stability, maintaining electrode functionality for 7 days at 4 °C and up to 60 days post‐fabrication (**Figure** **7**a). Nafion‐COF electrodes outperformed COF‐only surfaces in ASO or anti‐miR conjugation via streptavidin‐biotin chemistry, enhancing electrical output and facilitating proton/cation transport (Figure 7b,c). The results confirm that Nafion, along with modified COF, acts as an ion exchange membrane, facilitating the transport of protons (H+) or other cations. This enhances the conductivity of the electrode and improves charge transfer processes.

PEDOT:PSS, a conductive polymer, further increased sensor conductivity, stability, and flexibility, amplifying CV signals and reducing overpotential for MB redox reactions (Figure 7b,c). Its porous structure expanded the electrode's effective surface area, enabling more MB‐tagged ASOs to hybridize with miRNAs, improving signal strength and reproducibility (Figure 7d). The combined Nafion‐PEDOT system ensured consistent electrochemical properties over multiple cycles, providing reliable detection of ultra‐low miRNA concentrations (picomolar levels). This PEDOT/Nafion‐COF‐Streptavidin‐ASO platform significantly enhanced electrochemical output, electron transfer, and sensitivity, making it a robust tool for salivary miRNA detection in mTBI patients. Tests in artificial saliva confirmed some matrix effects but maintained strong quantifiable signals (Figure 7e). This high‐performance platform demonstrates excellent potential for electrochemical salivary miRNA sensors.

### 2.7 mTBI Salivary miRNA Detection from mTBI Patient Samples

From previous UV‐Vis and fluorescence spectroscopic measurements with artificial saliva, it was evident that anti‐miR ASOs exhibit minimal background interference from salivary components. After establishing the electrochemical detection method, saliva samples were collected from mTBI patients and healthy individuals (denoted as negative samples). The miRNA was extracted from the saliva through the standard miRNA extraction protocol, where the miRNA concentration in saliva was concentrated tenfolds, which was accounted for while performing the calculations. The results observed from electrochemical sensors were compared with PCR data to validate the presence of miRNAs in those samples. The details of PCR primers are provided in **Table** **3**.

**Table 3:** Database of primer sequences being used for PCR.

miR	Corresponding Primer Sequence
hsa‐miR‐532‐5p	5′CAUGCCUUGAGUGUAGGACCGU
hsa‐miR‐151a‐5p	5′UCGAGGAGCUCACAGUCUAGU
hsa‐miR‐30a‐5p	5′UGUAAACAUCCUCGACUGGAAG
hsa‐miR‐181a‐5p	5′AACAUUCAACGCUGUCGGUGAGU
hsa‐miR‐708‐5p	5′AAGGAGCUUACAAUCUAGCUGGG
hsa‐miR‐148b‐3p	5′UCAGUGCAUCACAGAACUUUGU
hsa‐let‐7e‐5p	5′UGAGGUAGGAGGUUGUAUAGUU
hsa‐miR‐145‐5p	5′GUCCAGUUUUCCCAGGAAUCCCU
hsa‐miR‐320b	5′AAAAGCUGGGUUGAGAGGGCAA
hsa‐miR‐16‐5p	5′UAGCAGCACGUAAAUAUUGGCG

### 2.8 Sensitivity and Specificity in Saliva Samples

**Figure 8:**
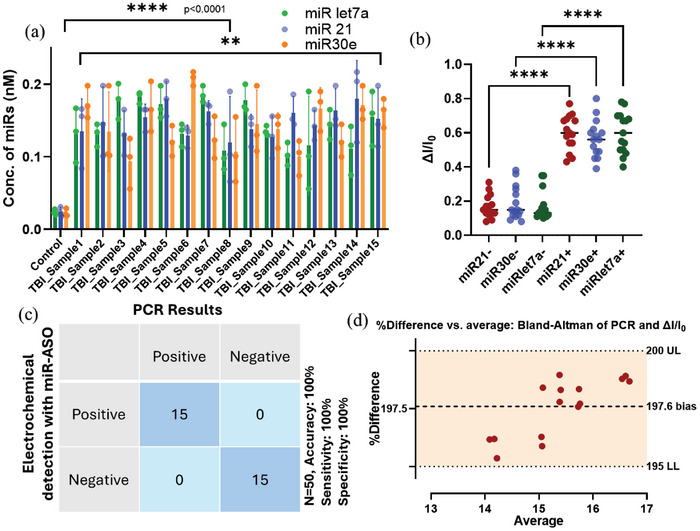
a) Concentrations of miRs in patients’ saliva samples incorporating calculation for concentration factor during miRNA extraction, depicted as per standard curve obtained from CV spectra for corresponding miR concentrations b) difference between miRNA levels for positive and negative patient samples c) confusion matrix for comparing electrochemical and PCR results d) Bland‐Altman plot for correlating the data obtained from electrochemical and PCT results.

Picomolar quantities of miRNA were successfully detected in saliva samples from patients with mild traumatic brain injury (mTBI). As miRNA extraction step concentrates the miRNA amount in samples, the picomolar concentrations in saliva were measured as nanomolar concentrations in the final extracted miRNA samples and the concentration factor was calculated. The concentrations of corresponding miRNAs were measured by cyclic voltammetry, and the values were calculated from previously obtained standard curves depicted for the corresponding miRNAs’ Cyclic voltammetry data (**Figure** **8**a). 2way ANOVA was performed for the grouped clinical samples. The electrochemical data correlated significantly between control and positive samples (****, *p* < 0.0001), validating the sensor's accuracy (Figure 8a).

The miR let7a, mir21, and miR30e positive samples showed significant differences with negative clinical samples when measured in cyclic voltammetry and calculated as ∆I/I_0_, where I_0_ was the initial current output before the addition of sample and ∆I is the current (I) difference between before and addition of the sample (Figure 8b). The sensor demonstrated 100% sensitivity and specificity, accurately assessing 30 patient samples (15 positive and 15 negative). The confusion matrix in Figure 8c shows the accuracy of the electrochemical detection and their correlation with PCR results. The Bland‐Altman plot analysis (Figure 8d) was performed between ∆I/I_0_ and PCR C_t_ values for the 15 positive mTBI clinical samples, and it was measured as % difference. The % difference was range bound within an upper limit of 200 and a lower limit of 195 for all the samples, which signifies the significance of the electrochemical detection compared to PCR results.

## 3 Conclusion

The successful detection of miR‐30e, miR‐21, and let‐7a in saliva samples suggests the potential for a multi‐marker panel to enhance diagnostic precision for mTBI. Given that additional miRNA biomarker candidates for mTBI exist, multiplexing our approach could provide significant diagnostic benefits.^[^ ^99^ ^]^ Post‐synthetic modifications of COFs could enable multi‐modal detection of miRNAs, further improving diagnostic capabilities. This study demonstrates the effective use of CV and DPV for detecting specific miRNAs using post‐synthetically modified COFs conjugated with MB‐labeled ASOs. The sensor's high sensitivity and specificity, validated against qPCR, highlight its potential for non‐invasive mTBI diagnostics. Future work will expand the range of detectable miRNAs and refine the sensor's specificity and sensitivity through further post‐synthetic modifications and control experiments.

## 4 Experimental Section

### Synthesis

1,3,5 triphenylphloroglucinol was synthesized following the protocol detailed by Chong et al.^[^ ^78^ ^]^ In a reaction vessel, hexamethylenetetraamine (15.10 g) and dried phloroglucinol (23.72 g) were combined under an inert nitrogen atmosphere. Trifluoroacetic acid (90 mL) was added, and the mixture was heated to 100 °C for ≈2.5 h. Subsequently, 150 mL of 3 m hydrochloric acid was introduced, and heating was maintained at 100 °C for an additional hour. After cooling to ambient temperature, the solution was filtered through Celite and extracted with dichloromethane (≈ 350 mL). The organic layer was dried over magnesium sulfate, filtered, and concentrated under reduced pressure to yield a proportionate amount of an off‐white powder. The product exhibited high purity as determined by ^1^H NMR and could be further purified by sublimation if desired.

### Powder X‐Ray Diffraction

Powder X‐ray diffraction (PXRD) was conducted using a Rigaku MiniFlex 600, scanning at a rate of 10° min^−1^ within the 2θ range of 3 to 40°, utilizing graphite monochromatized Cu Kα radiation (λ = 0.15405 nm).

### Scanning Electron Microscopy

Sample morphology was examined using a field‐emission scanning electron microscope (FE‐SEM, S‐4800, Hitachi) with an energy‐dispersive X‐ray (EDX) spectrometer. The COF and COF‐polymer‐based platforms were drop casted on the Au‐electrode, and the sensor strip containing the electrode was imaged before and after the addition of each layer and the electrodes are fixed on SEM stubs coated with double‐sided carbon tape, air‐dried, and then sputter coated with 80% platinum/20% palladium to achieve conductivity. The FEI Nova NanoSEM 450 was then used to monitor the surface topography of each of the samples. The field‐emission scanning electron microscopic images were recorded. The respective EDS spectra were also recorded for each of the samples.

### Raman Spectroscopy

Raman spectra were collected using a Renishaw inVia Reflex Raman Spectroscope system with the following parameters: 785 nm laser, 45 mW (50%) power, grating of 1200, 100× magnification, acquisition time 0.3 s with the center of Raman frequency set at 1100 cm^−1^.

### X‐Ray Photoelectron Spectroscopy

The samples were drop‐casted and vacuum‐dried each time to form a thick layer. XPS experiments used a Physical Electronics VersaProbe III instrument with a monochromatic Al kα X‐ray source (hν = 1486.6 eV) and a concentric hemispherical analyzer. Charge neutralization was performed using low‐energy electrons (<5 eV) and argon ions. The binding energy axis was calibrated using sputter‐cleaned Cu (Cu 2p3/2 = 932.62 eV, Cu 3p3/2 = 75.1 eV) and Au foils (Au 4f7/2 = 83.96 eV). Peaks were charge referenced to the CHx band in the carbon 1s spectra at 284.8 eV. Measurements were made at a takeoff angle of 45° concerning the sample surface plane. This resulted in a typical sampling depth of 3–6 nm (95% of the signal originated from this depth or shallower). Quantification was done using instrumental relative sensitivity factors (RSFs) that account for the X‐ray cross‐section and inelastic mean free path of the electrons. The analysis size was ∼200 µm in diameter. The analysis was performed in CasaXPS software.

### NMR Spectroscopy

NMR experiments were performed on a Bruker Avance II 300 MHz spectrometer (7.05T, 11B: 96.38 MHz). Single‐pulse excitation with a short rf pulse of 1 us was used to acquire the spectra. 4096 scans were collected with a relaxation delay of 4 s. All spectra were recorded at ambient temperature with 12 kHz magic angle spinning. The chemical shift was referenced to (δ = 0 ppm). Spectral line shape fits were calculated using the solid line shape analysis (SOLA) program in Topspin. The fitting model included chemical shift anisotropy and quadrupolar interaction for 4 different chemical sites. Topspin software was used for analyzing the data.

### Thermogravimetric Analysis

Thermogravimetric analysis (TGA) data were collected with a Thermal Analysis Instrument (SDT 2960, TA Instruments, New Castle, DE), operating at a heating rate of 10° min^−1^ under a nitrogen flow of 100 mL min^−1^.

### Fluorescence Microscopy

Images were obtained with a Zeiss LSM 880 confocal microscope. Fluorescence imaging of Cy3‐streptavidin was performed using Cy3 specific fluorescent channel.

### UV–vis and Fluorescence Spectroscopy

UV‐vis absorption spectra were measured with a Hitachi U‐3100 spectrophotometer. The total miR concentration was determined using Thermo Scientific NanoDrop OneC Microvolume UV–vis Spectrophotometer. The absorbance spectra for the assay with 96‐well plates were recorded on Biotek Synergy Neo2Microplate Reader for endpoint, kinetic, and spectral analyses. Each experiment was repeated at least three times and an average of these spectra were presented. Two samples from each category were selected randomly to standardize the assays. The absorbance spectra were then normalized. The highest absorbance value was chosen for each spectrum, and then the absorbance values were divided by that number. The normalized data was then compared to standardize the assay parameters, regardless of the details of the experiment. Fluorescence spectra were recorded in the solid and solution phases in the Horiba Fluromax plus fluorescence spectrophotometer.

### FTIR Measurements

Fourier transform infrared (FTIR) spectra were recorded using a Vertex PerkinElmer 580BIR spectrophotometer (Bruker) employing the KBr pellet technique.

### Transmission Electron Microscopy

Transmission electron microscopy (TEM) images were taken on an FEI Tecnai G2 S‐Twin equipped with a field emission gun operating at 200 kV. 20 µL solution of all the samples was added on top of a carbon‐coated copper grid (400 mesh). This was allowed to stay for ≈10 min before being removed with a filter paper and imaged under a transmission electron microscope (FEI tecnai T12). The tungsten filament with 80 kV accelerating voltage was used for the investigations.

### Electrochemical Measurements

The electrochemical measurements were performed using the electrochemical sensor interface PalmSens Sensit‐smart (Palm Instruments, The Netherlands), controlled by a PC running PSTrace software version 5.6 and mobile based android app PSTouch. The three‐electrode Zimmer & Peacock electrochemical cell (L4.5 ×W0.8 cm) has a gold working electrode. Each electrode is produced by screen‐printing technology and constitutes of a rectangular gold working electrode (3 mm diameter), a silver pseudoreference electrode and a graphite counter electrode.

The difference in charge transfer capacitance (electrochemical potential difference) of COF‐polymer‐ASO functionalized electrodes upon interaction with target miRNAs was measured by the portable device‐based biosensor. The DPV and CV parameters were optimized. CV data were recorded using a current range of 100 µA to 10 mA with a potential range of −0.7–0.7 V having step of 0.1 V and 3 number of scans were taken. For the DPV experiments the current range was selected from 1 µA to 10 mA. Potential range was set from ‐0.5 to 0.5 V with a step of 0.01 V.

Then, miRNAs were gradually added to it at different concentrations, and CV, DPV, and graphs were generated as standard curves. mTBI salivary samples were isolated through miRNA extraction and then drop‐cast over the functionalized electrodes.

## Conflict of Interest

SDH formerly served as the chief medical officer and a paid scientific advisory board member for Quadrant Biosciences, as well as a scientific advisory board member for Spectrum Solutions. SDH is named as a co‐inventor on a patent involving the use of salivary miRNA for clinical management of concussion, which is assigned to the Penn State College of Medicine. Prof. Pan is the founder or co‐founder of 5 start ups. None of these entities, however supported this work.

## Author Contributions

D.S., T.A., P.M., M.A., and K.D. contributed equally to this work. D.P. conceived the idea. P.S., P.M., M.A., K.D., and D.P. designed the study. P.S. and D.S. synthesized and characterized the compounds. P.S., T.A. and D.S. performed the experiments. M.M. helped P.S. in experiments. P.S. and D.P. analyzed the data and wrote the manuscript. All the authors approved the final draft of the manuscript.

## Supporting information



Supporting Information

## Data Availability

The data that support the findings of this study are available from the corresponding author upon reasonable request.
