# Supplementary material for: Tailored Anti‐miR Decorated Covalent Organic Framework Enables Electrochemical Detection of Salivary miRNAs for Mild Traumatic Brain Injury
Source: Small. 2025 Feb 17;21(14):2412107. doi: 10.1002/smll.202412107 (PMC11983262; doi:10.1002/smll.202412107)
Supplement: Supplementary file 1 — Supporting Information [file SMLL-21-2412107-s001.docx]

**Supporting Information**

**Tailored Anti-miR Decorated Covalent Organic Framework Enables Electrochemical Detection of Salivary miRNAs for Mild Traumatic Brain Injury**

*Pranay Saha^a^, David Skrodzki^b^,† Teresa Aditya^a^,† Parikshit Moitra^a,α^,‡ Maha Alafeef^a,c^,‡ Ketan Dighe^d^,‡ Matthew Molinaro^e^, Steven D. Hicks^f^ and Dipanjan Pan^a,b,g,h^*

^a^Department of Nuclear Engineering, The Pennsylvania State University, University Park, PA 16802, USA

^b^Department of Materials Science and Engineering, The Pennsylvania State University, University Park, PA 16802, USA

^c^Biomedical Engineering Department, Jordan University of Science and Technology, Irbid 22110, Jordan.

^d^Department of Biomedical Engineering, The Pennsylvania State University, University Park, PA 16802, USA

^e^Department of Engineering Science and Mechanics, The Pennsylvania State University, University Park, PA 16802, United States

^f^Department of Pediatrics, Penn State Health Children’s Hospital, Hershey, PA 17033, USA

^g^Huck Institutes of the Life Sciences, 101 Huck Life Sciences Building, University Park, PA 16802, USA

^h^Center for Infectious Disease Dynamics, The Pennsylvania State University, University Park, PA 16802, United States

^α^Current address: Department of Chemical Sciences, IISER Berhampur

†,‡ Authors contributed equally


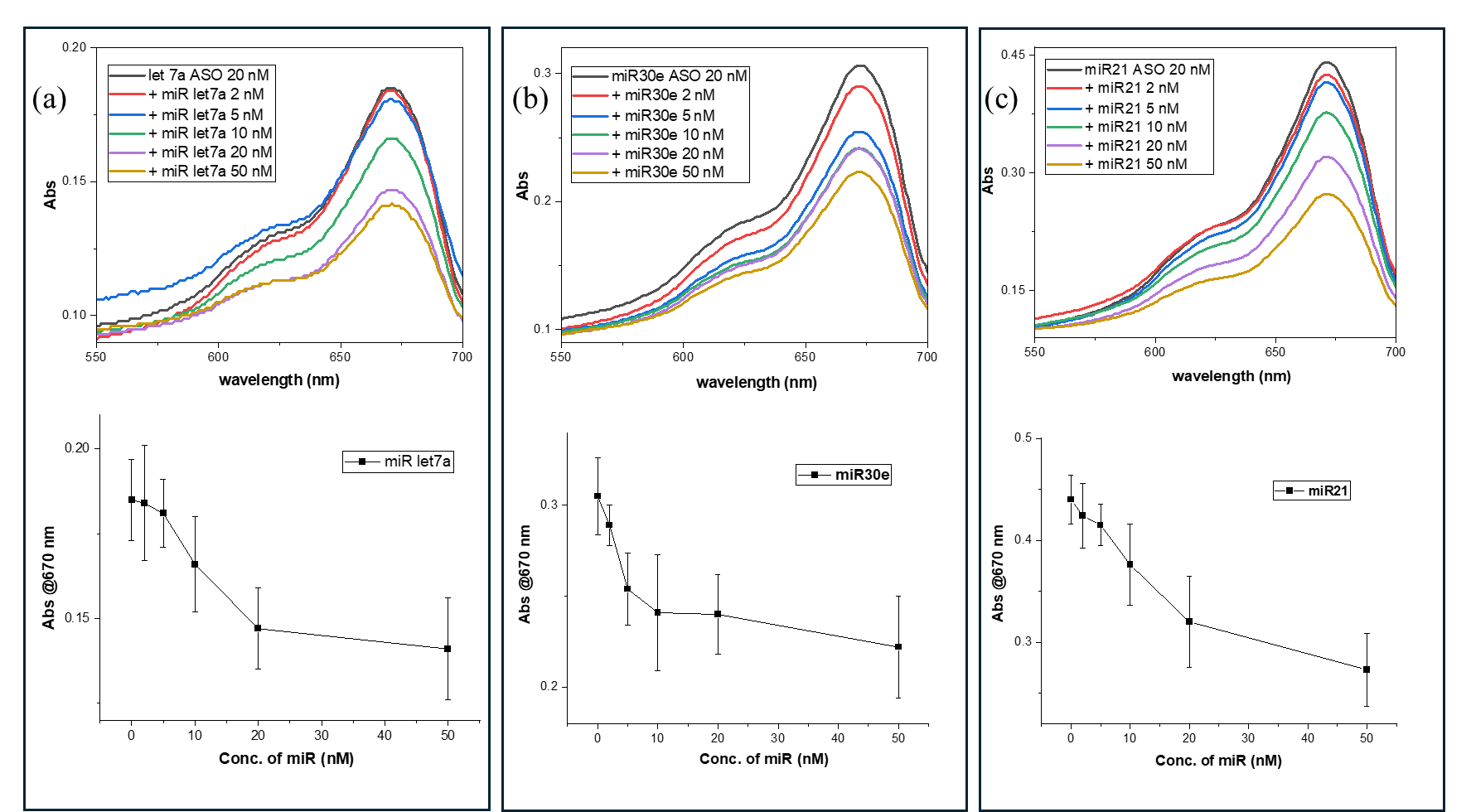


**Figure S1.** Change in absorbance of Methylene Blue upon increasing concentrations of miRNAs in aqueous solutions shows linear decrease in absorbance due to hybridization of complimentary ASOs and miRs.

**Figure S2.** Raman spectroscopy shows changes in Raman peaks upon hybridization of miR ASO with miR on Au electrodes


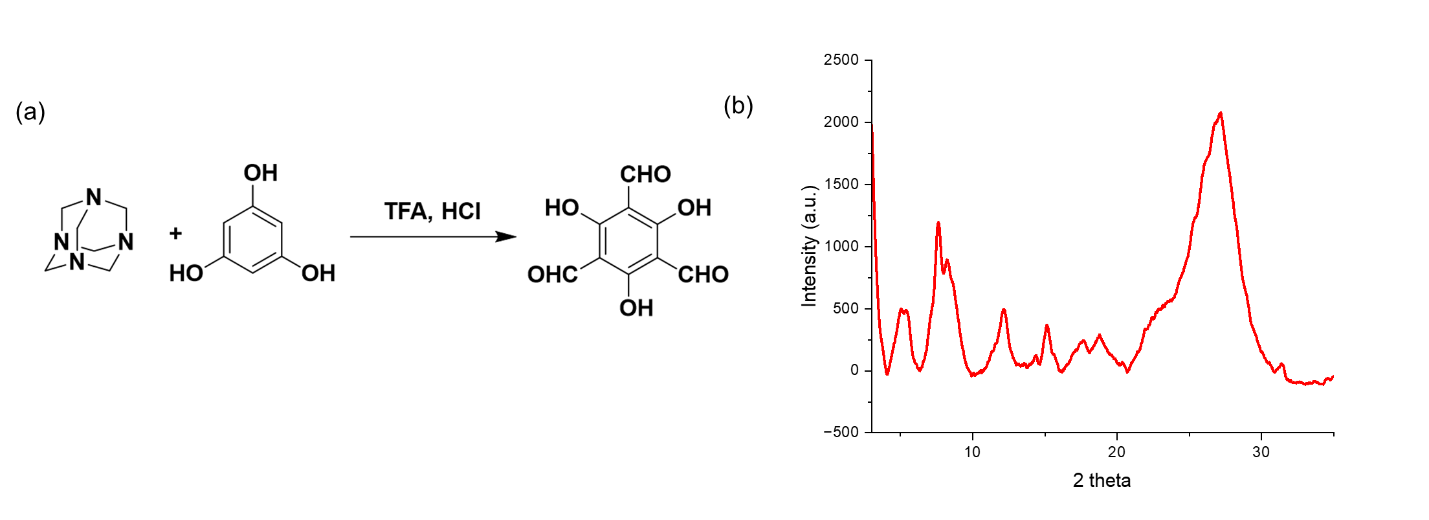
**Figure S3.** (a) Synthesis of 1,3,5-triformylphloroglucinol (b) XRD of p-phenylenediamine-1,3,5-triformylphloroglucinol COF shows clear crystalline peaks

**
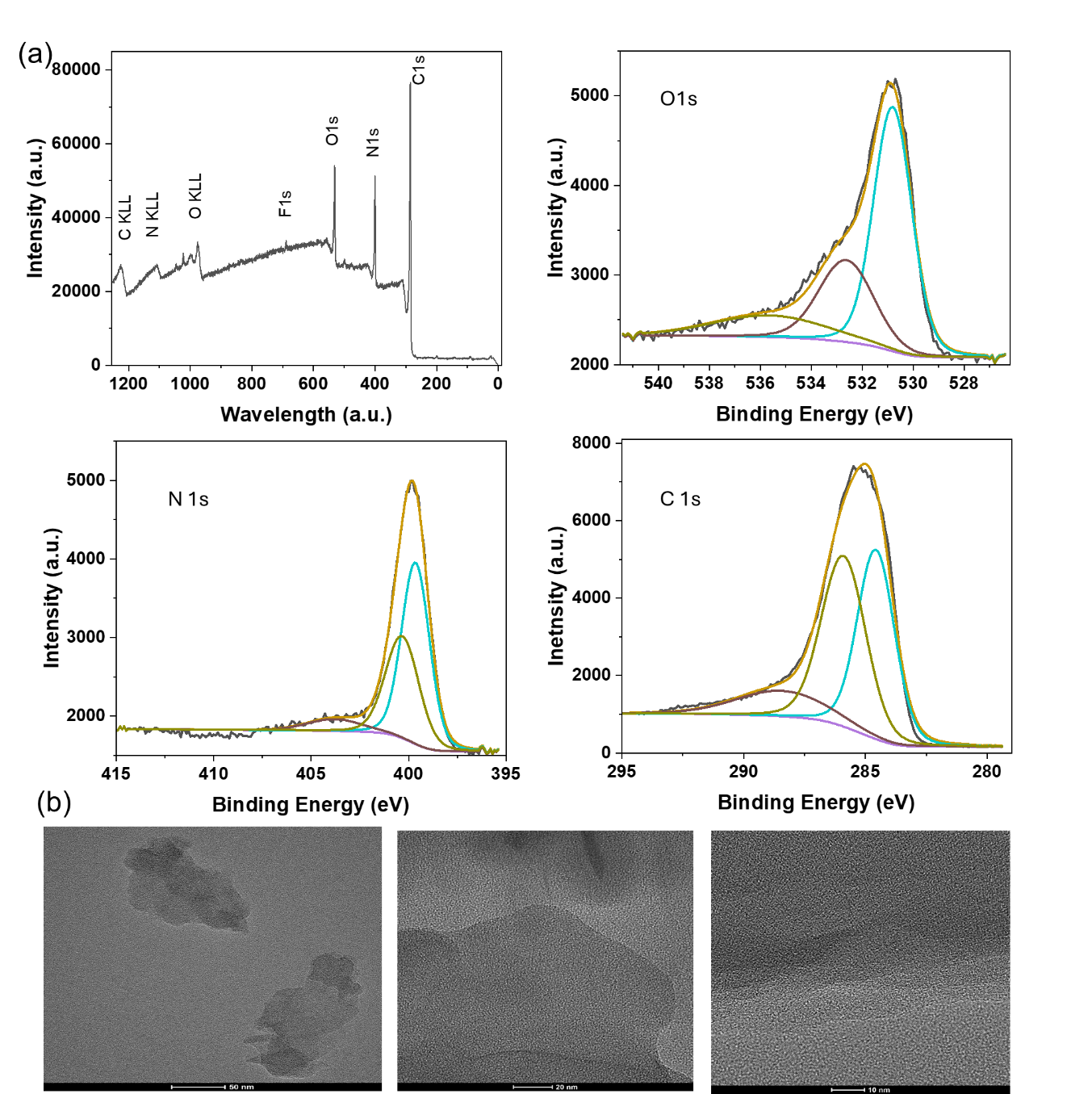
**

**Figure S4.** (a) XPS data shows the binding energies of electrons from different orbitals in the covalent organic framework. (b) Transmission electron microscopic images show the monolayer and multilayer accumulation of COF nanosheets with faintly visible lattice fringes.


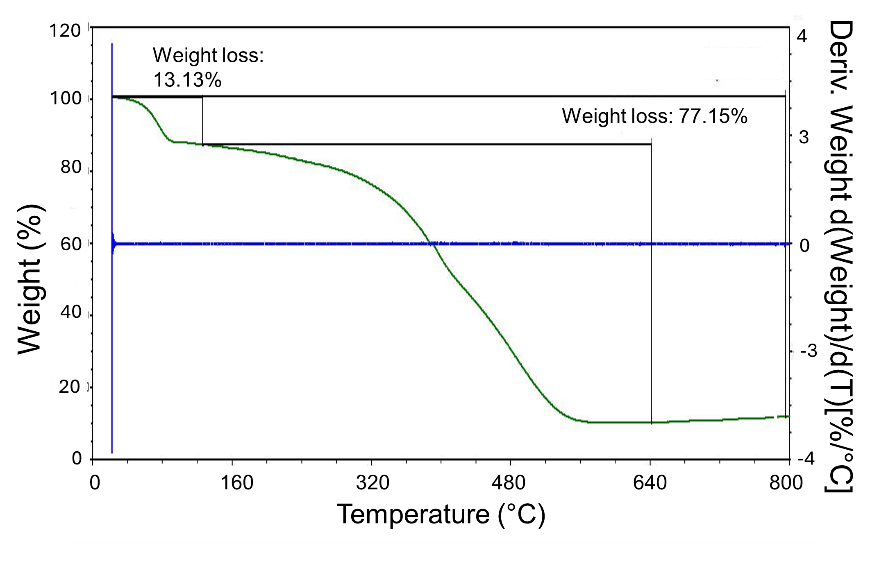
**Figure S5.** Thermogravimetric analysis of silane-modified COF shows gradual weight loss from 140°C to 520 °C

**
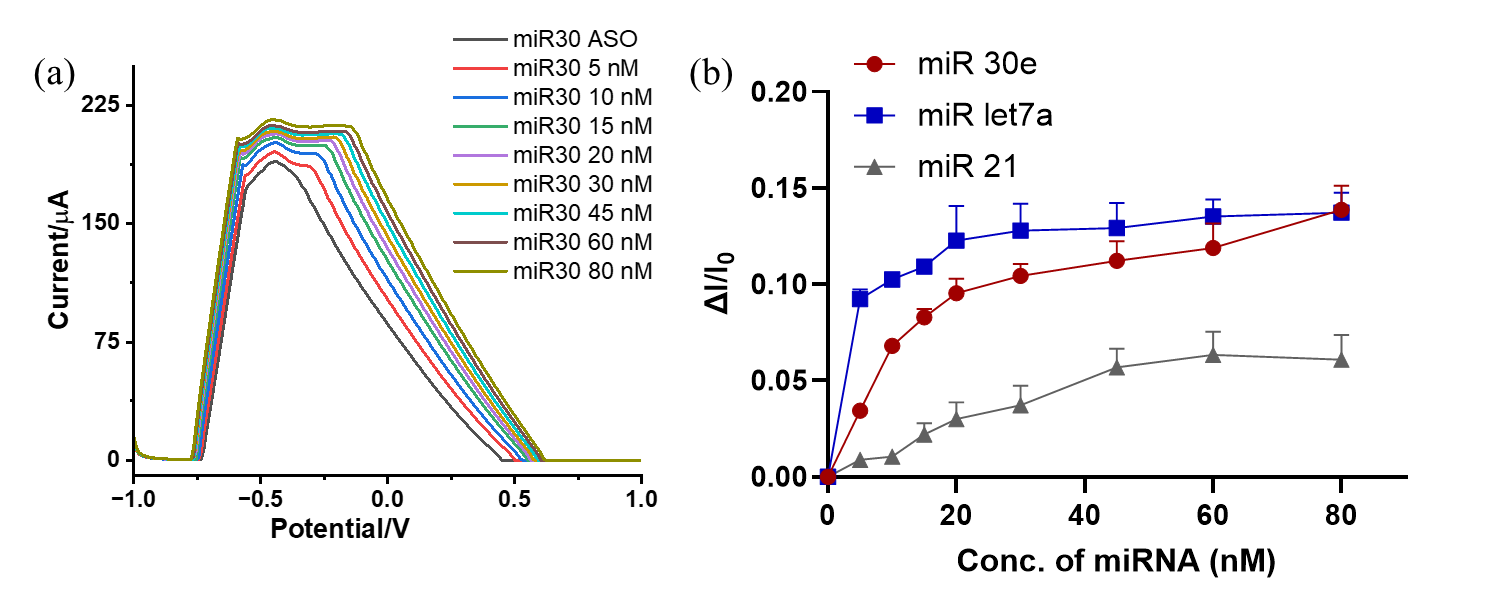
**

**Figure S6.** Differential pulse voltammetry (DPV) plots for (a) miR 30 and (b) comparative change in current in DPV for all three miRNAs upon hybridization with their corresponding anti-miRs functionalized over Au-electrodes
